# An Extensive Syncopal Workup for Geriatric Patients After a Traumatic Fall: Is It Worth It?

**DOI:** 10.7759/cureus.87190

**Published:** 2025-07-02

**Authors:** Shirin Siddiqi, Shawna Morrissey, Thomas Simunich

**Affiliations:** 1 General Surgery, Conemaugh Memorial Medical Center, Johnstown, USA; 2 Trauma and Acute Care Surgery, Conemaugh Memorial Medical Center, Johnstown, USA; 3 Research & Performance Excellence & Quality, Conemaugh Memorial Medical Center, Johnstown, USA

**Keywords:** geriatric fall, geriatric trauma management, patient outcomes, syncopal episode, traumatic fall, unexplained syncope

## Abstract

Background: Identifying the cause of syncope in geriatric patients presenting with syncope causing a traumatic fall is challenging in the absence of well-established guidelines. We hypothesize that most of the geriatric inpatient syncopal workup, including transthoracic echocardiogram and carotid duplex, is excessive and can be safely abandoned.

Methods: A retrospective review of data collected on 134 patients from Conemaugh Memorial Hospital, a single-level I trauma hospital in Johnstown, Pennsylvania, from January 2017 to October 2021 was carried out. Patients ≥65 years old, with a diagnosis of syncope and fall with a Glasgow Coma Scale ≥14, were included. Demographics, initial inpatient syncopal workup, including cardiac monitor, electrocardiogram, cardiac enzymes, orthostatic vitals, transthoracic echocardiogram, carotid duplex ultrasound, and thyroid-stimulating hormone levels, were recorded. Intervention, including Holter monitoring, cardiac catheterization, or implantation of a defibrillator, was also captured. Measurement of outcomes was presented as counts and percentages.

Results: A total of 747 studies were done for 134 patients, out of which 17 studies (2.3%) for 9% (12/134) of patients had positive findings. This translated into 6% (8/134) of patients requiring a change in treatment based on those findings.

Discussion: Based on our results, most causes of syncope can be diagnosed with cardiac monitoring and an ECG. Cardiac monitoring and an electrocardiogram were most likely to yield a positive finding. Further testing with transthoracic echocardiogram (TTE) or carotid duplex can be safely performed on a case-by-case, outpatient basis.

Conclusion: Syncopal workup should be ordered based on the initial assessment, review of the pre-hospital medications, and ECG findings. ​Additional testing can be performed on a case-by-case, outpatient basis, depending on the physician’s discretion for more efficient care and a reduction in healthcare expenditure.

## Introduction

Syncope, defined as a transient loss of consciousness and postural tone followed by spontaneous recovery, is responsible for 1-3.5% of all emergency department (ED) visits and 6% of all hospital admissions in the United States [1]. Syncope has multiple causes, ranging from benign to life-threatening. While the American College of Cardiology, the American Heart Association, and the Heart Rhythm Society have published guidelines for the evaluation and management of patients with syncope, these guidelines are not specific to geriatric patients who have experienced a traumatic fall. Hence, identifying the cause of syncope in geriatric patients presenting with syncope causing a traumatic fall is challenging in the absence of well-established, evidence-based management guidelines. This often leads to an extensive inpatient workup, including carotid duplex, transthoracic echocardiogram (TTE), head computed tomography angiography (CTA), or magnetic resonance imaging (MRI), despite rarely affecting medical management (<5 %) [1]. The combination of high hospital admissions and multiple diagnostic tests with low diagnostic yield contributes to an increase in hospital length of stay (LOS) and hospital costs, estimated to be $5,300 per admission and exceeding $2.4 billion annually in the United States [2,3]. With no change in medical management for ~95% of these cases, nearly all inpatient syncopal workups yield medically inconsequential results and are therefore more appropriately performed on an outpatient basis. Based on our literature search targeting level 1 trauma centers nationwide, we hypothesize that most geriatric inpatient syncopal workups, including TTEs and carotid duplexes, are excessive and can be safely abandoned.

## Materials and methods

This retrospective study was conducted at Conemaugh Memorial Hospital, a single-level I trauma hospital in Johnstown, Pennsylvania, with data collected from ED presentations between January 2017 and October 2021. The study was determined to be exempt from the Institutional Review Board review. Patients ≥65 years old, who presented to the ED with Glasgow Coma Scale (GCS) ≥14, and who were diagnosed with both syncope and fall upon presentation were included in the study. Patients with seizure disorder or drug/alcohol intoxication upon presentation were excluded. 

Electronic medical records were used to identify demographics, including age, gender, injury severity score, and comorbidities. ICD codes were used to identify patients presenting with both syncope and fall. Diagnostic tests obtained as part of initial syncopal workup included cardiac monitor, 12-lead electrocardiogram (ECG), cardiac troponin (0.00-34.00 ng/L per the hospital’s reference value), orthostatic vitals, TTE, carotid duplex ultrasound, thyroid-stimulating hormone (TSH) levels (0.35-4.94 IU/mL per the hospital’s reference value), and electroencephalogram (EEG). An abnormal finding on cardiac monitor or 12-lead ECG was defined as arrhythmia, atrioventricular nodal block, heart rate <40 beats per minute, pacemaker malfunction, QRS complex duration >100 milliseconds, QT interval >450 milliseconds in males or >470 milliseconds in females, sinus pause >3 seconds, ST changes, or pathologic Q waves. An abnormal TTE was defined as a left ventricular ejection fraction <50%, valvular abnormalities, cardiac tamponade, wall motion abnormality, or pulmonary hypertension. 

The number of consultations from cardiology, neurology, or vascular surgery was captured. Records were reviewed to determine whether an intervention was performed and to identify the elements in the workup that led to it. Additional workups performed while the patient was admitted to the hospital included Holter monitoring, cardiac catheterization, and placement of a permanent pacemaker (PPM) or automatic implantable cardioverter-defibrillator (AICD). Lastly, any outpatient workup after discharge was also recorded. The cause of syncope was categorized as cardiogenic, neurogenic, other (including vasovagal syncope), or undetermined. Measurement of outcomes were counts and percentages (e.g., studies with positive finding(s) / total studies done; treatment change due to positive study finding(s)/total studies done; studies with positive finding(s)/total unique patient encounters; and treatment change(s) due to positive study finding(s) / total unique patient encounters).

## Results

During the four-year study period, 134 patients with an average age of 81 years were included in the study; 61.5% were females (Table 1).

**Table 1:** Characteristics of the study patients (N = 134) The data are represented as N, %, and mean ± standard deviation (SD).

	Female	Male	Overall
Sex	60.4% (81/134)	39.6% (53/134)	----
Mean age (years) ± SD	83 ± 8.2	79 ± 8.9	81 ± 8.8
Mean length of stay, (days) ± SD	4.2 ± 2.5	4.5 ± 3.7	4.3 ± 3.0
Comorbidities			
Hypertension	79% (64/81)	73.6% (39/53)	76.9% (103/134)
Coronary artery disease	38.3% (31/81)	37.7% (20/53)	38.1% (51/134)
Diabetes	35.8% (29/81)	35.8% (19/53)	35.8% (48/134)
Hyperlipidemia	39.5% (32/81)	26.4% (14/53)	34.3% (46/134)

**Figure 1:**
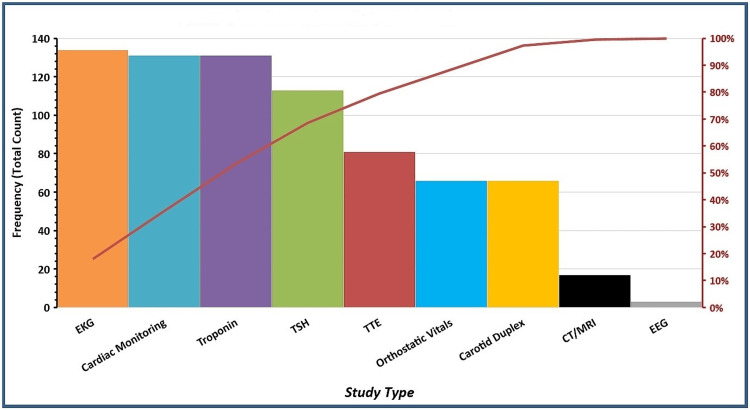
Pareto of studies performed on the initial work-up by study type EKG: electrocardiogram, TTE: transthoracic echocardiogram, TSH: thyroid-stimulating hormone, EEG: electroencephalogram, CT: computed tomography, MRI: magnetic resonance imaging.. The data are presented as %.

The most common comorbidities were hypertension (103 patients, 76.9%), coronary artery disease (51 patients, 38.1%), diabetes mellitus (48 patients, 35.8%), and hyperlipidemia (46 patients, 34.3%). The average hospital length of stay (LOS) was 4.3 days. A total of 747 studies were done for 134 patients, out of which 2.3% of studies had positive findings. This translated into 6% of patients requiring a change in treatment based on those findings (Figure 1).

Of the three specialties we reviewed, cardiology was consulted for 60 (44.8%) patients, and neurology was consulted for 10 (7.5%) patients. Only two (3.3%) patients on whom cardiology was consulted were found to have a cardiac cause of syncope. Vascular surgery was only consulted on 1.5% patients. On those two patients, one had chronic, stable right internal carotid artery stenosis that did not require any intervention. The other patient was prescribed aspirin (acetylsalicylic acid) 81 mg for bilateral carotid artery stenosis. 

The cardiac monitor showed arrhythmia in 19 (14.1%) patients. Twenty-four (17.9%) had positive findings on 12-lead ECG (prolonged QT interval). Sixteen (11.9%) patients had positive orthostatic vitals. Cardiac troponins were found elevated in 3.7% patients, but serial troponins were stable and attributed to trauma instead of cardiac ischemia. Fifty (37.3%) patients were found to have left ventricular ejection fraction (LVEF) <50% on TTE, and 15/134 (11.1%) patients had pulmonary hypertension. However, none of these findings required further workup by the cardiology team, as only five patients had new but mild LVEF reduction. A total of 71 TTEs were ordered for our study population, none of which yielded information that altered the course of management. Five (3.7%) patients required a permanent pacemaker or automatic implantable cardioverter defibrillator (AICD). Of the five patients, two had arrhythmia, one had type II AV block, one had bradycardia, and one had sinus pause lasting >3 seconds found during initial cardiac monitoring and/or ECG. One of the five patients with bradycardia was also diagnosed with pulmonary hypertension on TTE. 

A total of 68 carotid duplex ultrasounds were obtained. Forty-seven (35%) patients had mild (< 49%), 15 (11.1%) had moderate, and one patient had severe (> 70%) carotid artery stenosis. The patient with severe carotid artery stenosis was started on aspirin and referred to outpatient follow-up with vascular surgery. 

Three (2.2%) patients were found to have acute to subacute intracranial hemorrhage on CTA head, with two of them being diagnosed with cardiogenic cause of syncope, while one remained undetermined. 

Out of 134 patients, 118 had normal TSH levels. Patients with abnormal TSH values were referred to their primary care physician for management. Three (2.2%) patients had EEG, none of which demonstrated seizure activity, and the cause of syncope remained undetermined.

A total of 100/134 (74.6%) patients had an undetermined cause of syncope. These 100 patients underwent 553 studies. Twenty-four (17.9%) patients had 145 studies and were found to have had a cardiogenic cause of syncope. One patient with cardiogenic cause of syncope with history of aortic stenosis required cardiac catheterization during the same admission to evaluate for worsening aortic stenosis, without significant findings. Three patients were discharged with a 30-day Holter monitor but were lost to follow-up. Five patients had changes to their medication. Hence, a total of 8/134 (6%) patients required any change in treatment based on the tests. Ten (7.4%) patients had syncopal falls attributed to orthostatic hypotension, vasovagal syncope, or neurogenic cause (including autonomic dysfunction due to Parkinson's disease and bilateral frontal lobe infarcts).

## Discussion

Syncope is a potential preventable cause of traumatic falls in geriatric patients. In this retrospective study, we identified 134 geriatric patients admitted over a four-year period who underwent a syncope workup after a fall. The overall workup led to an intervention in only 6% (8/134) of the patients. For five of the eight patients who had a PPM or AICD implanted, the need for intervention was recognized solely from initial cardiac monitoring and ECG results. Our results are consistent with the current literature. Maung et al. [1] found that on 300 patients >50 years of age who were admitted after a traumatic fall or motor vehicle collision (MVC) with suspected syncope, <5% of diagnostic testing with cardiac enzymes, echo and carotid duplex yielded clinically relevant information that led to an intervention (medication adjustment, specialty consult, permanent pacemaker, or automatic implantable cardioverter defibrillator). In 69% of those who underwent intervention, the intervention was initiated based on the initial assessment and did not depend on the results of subsequent diagnostic testing.

Diagnostic tests for syncope contribute to increasing health care expenditure. Our results demonstrate that routine TTE or carotid duplex ultrasound has a low yield of diagnostic information and rarely leads to a change in medical management. At our institute, the cost of each TTE and carotid duplex is approximately $1,700 and $850, respectively (Table 2).

**Table 2:** Costs of diagnostic tests at our institute in the evaluation of syncopal fall (cost in dollars)

Test	Cost ($)
Electrocardiogram (ECG)	33
Troponin	99
Thyroid-stimulating hormone (TSH)	108
Transthoracic echocardiogram (TTE)	1700
Carotid duplex	858
Computed tomography (CT) / magnetic resonance imaging (MRI)	~1600-4000
Electroencephalogram (EEG)	687
Specialty consult	~200-350

The cost per test affecting diagnosis or management was the highest for TTE ($137,700), carotid duplex ($58,344), and cardiac enzymes ($12,969). Our results are consistent with the published literature. Mendu et al. [4] conducted a study on 2,106 geriatric patients with syncope to determine the frequency, yield, and costs of inpatient workup. His results show that cardiac enzymes, CT scans, echocardiograms, carotid ultrasounds, and EEGs determine the etiology of syncope less than 2% of the time, with EEG, CT, and cardiac enzymes being the most expensive tests in the study population. 

Bhat et al. [5] conducted a retrospective study on 5,420 patients with traumatic falls, out of which 180 patients were also found to have syncope. They reported a diagnostic yield of 10.1% for continuous ECG monitoring via telemetry/Holter studies, 0.7% for echocardiography, and 0% for carotid duplex ultrasound. Similarly, Bandy et al. [6] conducted a retrospective study of 50 patients with an average age of 74 years and a GCS of 15 who presented to the ED after a fall and underwent transthoracic echocardiography and carotid duplex as part of the syncope workup. The study reported that only one patient had a lesion on echo (apical akinesia) that was thought to be a potential cause of syncope, who was referred for PPM on an outpatient basis. One of the five patients in our study who underwent PPM placement had a history of paroxysmal atrial fibrillation, with new findings of bradycardia, elevated troponin (serial troponin negative), and mild pulmonary hypertension found on TTE. However, the cause of syncope was attributed to symptomatic bradycardia, found on cardiac monitoring. Morrison et al. [7] reviewed 88 patients at a level 1 trauma center and concluded that a thorough history, physical examination, and admission laboratory values were the most helpful tools in diagnosis 59% of the time, with none of the patients with normal admission EKG found to have a cardiac cause of syncope. 

According to our results, of the 68 carotid duplexes ordered, only one resulted in a new positive finding of severe carotid artery stenosis. The patient was prescribed 81 mg of aspirin and referred to the vascular surgery outpatient. This is similar to a study by Harfouche et al. [8] comprising 736 trauma patients who presented after a fall or MVC, reported that 1.7% had severe carotid stenosis on carotid duplex ultrasound, but none underwent carotid endarterectomy/stent. Head CT, carotid US, and EEG are all known to rarely identify lesions contributing to syncope [4], and our results confirmed this. 

For 74.6% of our study patients, the cause of syncope remained undetermined. This number is higher than that reported in prior studies [4,5] and emphasizes that extensive workup provides low diagnostic yield.

Strengths and limitations 

Although this is a single-center, retrospective study, with reliance on electronic medical records with potential for missing data, our study affirms the compelling evidence against extensively aggressive and often unnecessary workup for patients with syncope and falls. However, recent literature does not contain any studies specific to the geriatric population (age ≥65 years), and to the best of our knowledge, this is the second study after Mendu et al. [4] to target this vulnerable population, in whom syncopal falls are associated with devastating morbidity and mortality. 

Another limitation is the lack of standard criteria for ordering tests; other than EKG and cardiac monitor, most of the other tests were attending physician/subspecialty specific. Hence, our study’s results need to be reproduced by performing a larger-scale, multicenter study on the geriatric population presenting with syncopal falls to create criteria and determine the cause using appropriate tests to optimize care. 

## Conclusions

Given the lack of a standardized syncope evaluation pathway, inpatient syncopal workup should be ordered based on the initial assessment, review of the pre-hospital medications, and ECG findings. The initial assessment should not be limited to the injuries upon presentation only, but should include all relevant medical history, risk factors, and signs and symptoms of neurologic and cardiovascular diseases that may then require further testing. Additional testing can be performed on a case-by-case, outpatient basis, depending on the physician’s discretion. This will allow more efficient care and a reduction in healthcare expenditure.
